# *Amanita phalloides*-Associated Liver Failure: Molecular Mechanisms and Management

**DOI:** 10.3390/ijms252313028

**Published:** 2024-12-04

**Authors:** Tahrima Kayes, Vincent Ho

**Affiliations:** 1 Department of Gastroenterology and Hepatology Campbelltown Hospital, Campbelltown, NSW 2560, Australia; v.ho@westernsydney.edu.au; 2 School of Medicine, Western Sydney University, Campbelltown, NSW 2560, Australia

**Keywords:** *Amanita phalloides*, mushroom poisoning, toxicity, α-amanitin, liver failure, hepatotoxicity, silibinin, liver transplant

## Abstract

*Amanita phalloides* is well-established as one of the most poisonous mushrooms; toxicity from ingestion was reported as early as the first century. Although native to Europe, this ectomycorrhizal fungus has been widely spread and is responsible for liver toxicity in many parts of the world. Toxicity is characterized by delayed gastrointestinal symptoms mimicking acute gastroenteritis followed by severe hepatotoxicity and liver failure with consequent multi-organ failure. The primary mechanism of liver toxicity is considered to be the inhibition of RNA polymerase II with consequent hepatocyte apoptosis. Treatment measures include supportive measures such as rehydration and correction of electrolytes on initial presentation, activated charcoal and lavage to decrease absorption, extracorporeal purification methods such as plasmapheresis, fractionated plasma separation and adsorption, and molecular adsorbent recirculating system, as well as drug therapies including antibiotics, N-acetylcysteine, and silibinin. Liver transplantation is required in those with acute liver failure and poor prognostic features. Here, we reviewed the basic biology, pathophysiology, and molecular mechanisms of *Amanita phalloides* liver toxicity, as well as available treatments.

## 1. Introduction

Mushroom consumption by humans and associated toxicity have been known for millennia, dating as early as the Roman Empire [1]. Over time, with advances in modern analytic methods such as chromatography and spectrometry, our knowledge and understanding of the characteristics of toxic mushrooms have led to the ability to isolate the causative compounds [1,2]. *Amanita phalloides*, commonly referred to as the “death cap” mushroom, is often involved in incidences of fatal mushroom poisoning in humans, most commonly manifesting in acute liver failure [2,3]. Broadly, there are two main groups of toxins in this species: amatoxins and phallotoxins [1,3]. Hepatotoxicity is primarily driven by amanitins, particularly α-amanitin. Clinical manifestations of *A. phalloides* intoxication are evident after a few hours’ incubation, primarily as gastrointestinal symptoms, including nausea, vomiting, diarrhea, and jaundice [3,4,5]. Early detection and recognition are important but often difficult, as symptoms may be presumed secondary to gastroenteritis [4]. Acute liver failure with sudden derangement in transaminases and jaundice are the main pathophysiologic features of amatoxin intoxication. There are no established treatment criteria, and supportive management is often attempted. However, a majority of patients proceed to emergent liver transplant, which has recognized survival benefits in this setting [5].

Here, we examine the biology of *A. phalloides*, molecular mechanisms of toxicity, clinical manifestations, and management of patients with *A. phalloides*-associated hepatotoxicity.

## 2. Biology of Amanita phalloides

### 2.1. Biology

The *Amanita* genus contains 900–1000 mushroom species and is mostly responsible for human poisoning after mushroom consumption [6]. Defining features of this family include stem tissue with vertically aligned inflated cells and gills with bilateral lamellar trama. *Amanita* mushrooms are very recognizable, with *Amanita muscaria*, the type species, being well-established in popular culture [6]. Representations of the distinct red cap and white spots of *A. muscaria* can be found in children’s literature and video games such as Super Mario [7]. The poisonous qualities of *A. phalloides* have likely been known since the Roman Empire, with the death of Emperor Claudius (AD 53) suspected to be caused by the consumption of food containing mushrooms—presumably, *A. phalloides* [1,8]. Despite extensive historical knowledge of poisonous mushrooms, cases of fatal mushroom poisoning still occur throughout the world, most commonly occurring after the ingestion of *Amanita* species, primarily *A. phalloides* [1,9].

Unlike the striking appearance of *A. muscaria* with a large red and white flat cap, *A. phalloides* has a smooth greenish-yellow cap that is easily peeled. The stalk, which is approximately 6–12.5 cm high, is smooth and white. An irregular ring near the top of the stalk and a bulbous cup at the base are features used to distinguish *A. phalloides* from other species, such as *Amanita virosa*, which also has a smooth white cap that turns yellow as it matures [10]. Like other Amanita species, the fruiting body has a sweet fragrance [11]. The toxic components of *A. phalloides* are amatoxins, primarily amanitins. It also produces phallotoxins, which have minimal toxic effects after oral ingestion [9,12,13]. Despite some distinct features, *A phalloides* are often mistaken for edible mushrooms such as *Volvariella volvacea* and *Amanita calyptoroderma*, leading to accidental ingestion [6].

### 2.2. Habitat

Although native to Europe, cases of *A. phalloides* poisoning have been reported throughout the world, including Australia, United States, Central and South America, Asia, and Africa [3,10,11,14]. *A. phalloides* is ectomycorrhizal and forms symbiotic relationships with different tree species including oak, chestnut, and pine enabling the introduction of the fungus to other parts of the world where these trees have been introduced [12,13,14]. They form symbiotic associations with the roots of these woody plants, providing soil nutrients in exchange for carbon from the host plants. Fruitification occurs during spring, late summer, and autumn, and intoxication often occurs during these times [14].

### 2.3. Toxins of Amanita phalloides

The toxic components of *Amanita* mushrooms were first noted early in the twentieth century in Baltimore when researchers were studying the properties of the similarly poisonous *Amanita virosa* [1,9]. Almost all our knowledge of the chemistry of *Amanita* toxins is from Wieland and colleagues in Germany [6,13]. The amatoxins are bicyclic octapeptides, and phallotoxins are cyclic heptapeptides, both with molecular weights of approximately 900 g/mol [1,9]. Mortality in mice via injection was how the toxic principles of *A phalloides* were established, with amatoxins being “slow-acting” and phallotoxins “fast-acting” toxins [6]. The amanitins are highly toxic, with a median lethal dose (LD50) of 0.3 to 20 mg/kg, leading to death in 2–10 days. Phallotoxins are lethal within 2–7 h, with an LD50 of 2 ± 3 mg/kg when injected, and have minimal toxic effects with oral exposure [1,9].

**Figure 1:**
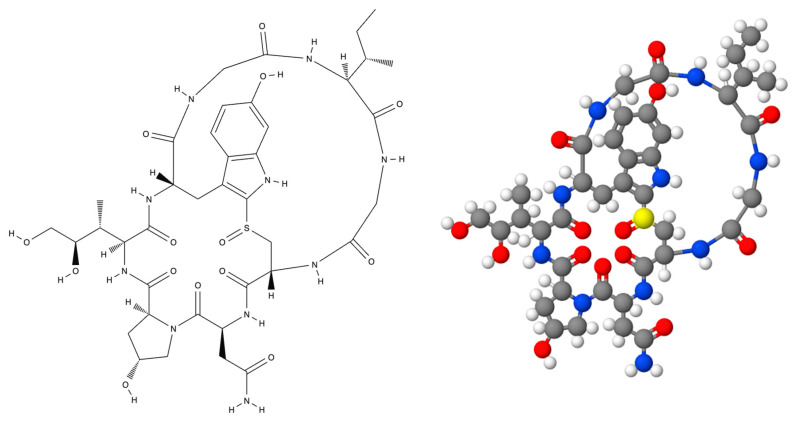
Molecular structure of α-amanitin.

The amatoxins are eight amino-acid residues organized in a conserved structure [9]. There are nine different amatoxin compounds: α-amanitin (Figure 1), β-amanitin, γ-amanitin, ε-amanitin, amanin, amaninamide, amanullin, amanullinic acid, and proamanullin. The molecular structures of this group of compounds only differ by number of hydroxyl groups and byanamide carboxyl exchange (Figure 1). The main toxicological studies have focused on α-amanitin and β-amanitin as they are present in greatest abundance at 66–75% and 24–32%, respectively, in the fruiting body and in all morphological fractions [1,9,13]. The toxins typically have an outer and inner loop consisting of eight amino acids. The outer loop is formed by peptide bonds between the C- and N-termini of the amino acids. The inner loop is created by the “triptathionine” bond between 6-hydroxytryptophan and cysteine [1,6]. The bicyclic ring structure plays an important role in activity as breaking the cycle at either the bond between Isoleucine #1 and Tryptophan #2 or between Tryptophan #2 and Cysteine #5 (the cross-bridge) destroys activity (Figure 1) [6].

Amatoxins have good heat stability and are not destroyed by cooking or drying [1,3,15,16]. They decompose slowly when stored in the open, in aqueous solutions, or following prolonged exposure to sunlight [3,17]. Indeed, when α-amanitin was stored in water at room temperature for 6 months, 86% of the toxin remained; when stored similarly in methanol, 96% of the toxin remained. Approximately 5% of the toxin remains after 6 h of cooking at boiling temperatures. However, changes in chemical structure with long cooking do not necessarily lead to potentially reduced toxic effects [1,18]. Baked mushrooms were noted to have higher concentrations of amatoxin compared with grilled or oil-fried mushrooms [18,19].

Amatoxins are soluble in water, methanol, and other organic solvents due to the presence of side-chain hydroxyl groups on the molecules [1,19]. Furthermore, the toxins are resistant to enzyme and acid degradation, which prevents breakdown during cooking or in the gastrointestinal tract. This is suspected to be due to the bicyclic peptide structure [1,3,20]. A case of fatal ingestion of *A. phalloides*, in which the victim consumed mushrooms that had been frozen for 7–8 months, suggesting stability to freezing and thawing processes, was reported [1,18].

Amatoxins have been detected in dried herbarium specimens that were at least 20 years old, further demonstrating their stability in cooking. The stability of the compounds in various temperatures, storage, and cooking methods strengthens the risk of poisoning from ingestion. *A. phalloides* are commonly mistaken for edible mushrooms [6,18], increasing the risk of food safety hazards and the need for public awareness to avoid consuming unidentified field mushrooms.

Phallotoxins consist of at least seven compounds (phalloidin, phalloin, prophallin, phallisin, phallacin, phallacidin, and phallisacin) that contain similar peptide rings [13]. Phallotoxins bind to F-actin, stabilizing the actin filaments, preventing microfilament depolymerization and disrupting the correct function of the cytoskeleton. Phallotoxins are only toxic to mammals if administered via a parenteral route since they are not absorbed through the gastrointestinal tract [3].

## 3. Mechanisms of Gastrointestinal and Liver Toxicity

### 3.1. Pathophysiology of Toxicity

The clinical spectrum of *A. phalloides* poisoning can range from mild subclinical presentation to a lethal fulminant course. Not all patients with *A. phalloides* poisoning develop acute liver failure or have fatal outcomes. After *A. phalloides* ingestion, there are four phases of poisoning: an asymptomatic latent or “lag” phase lasting 6–18 h after ingestion, a gastrointestinal phase occurring 6–24 h after ingestion characterized by symptoms of abdominal pain, nausea, vomiting, and watery diarrhea [2,11,16]. The clinical and metabolic consequences of these symptoms include hypotension, electrolyte disturbances, impaired renal function, and metabolic acidosis [5,10,11]. An apparent convalescence phase occurring 36–48 h after ingestion is characterized by a temporary resolution of gastrointestinal symptoms, but progressive hepatic impairment is evident with rising liver chemistry, coagulopathy, and jaundice [4,11,21].

The final phase is progression to acute liver failure with a dramatic rise in transaminases and bilirubin associated with coagulopathy, hypoglycemia, acidosis, renal failure (potentially hepatorenal syndrome), and hepatic encephalopathy [11,21]. Multi-organ failure, disseminated intravascular coagulation, rapid central nervous system deterioration, severe hemorrhagic manifestations, and death may occur within 1–3 weeks after ingestion. However, patients with favorable outcomes will see rapid improvement in liver chemistry with recovery and restoration of quality of life [10,15,22,23].

The mortality rate of *A. phalloides* poisoning is 10–20% [11]. Clinical parameters associated with worse outcomes include lower blood pressure at admission, higher baseline aminotransferases, bilirubin, and international normalized ratio (INR), and higher lactate, as well as more pronounced hyponatremia in comparison to patients with spontaneous recovery [4,23]. A small study has found all patients with mushroom poisoning who had bilirubin of >85 μmol/L and a partial thromboplastin time (aPTT) of >50 s on day 3 of admission died from the poisoning, suggesting these parameters may also indicate poor outcomes [24].

Liver biopsy findings of patients who have consumed *A. phalloides* vary depending on the extent of intoxication [23]. Biopsies of explanted livers have demonstrated massive centrilobular hemorrhagic necrosis and vacuolar degeneration of hepatocytes with significant fatty infiltration [3,22,23,25]. Steatosis often precedes the appearance of centrilobular necrosis [23]. In patients with moderate non-fatal poisoning, centrilobular necrosis without steatosis or the development of an initial inflammatory response has been noted. After a few weeks, phagocytosis of necrotic cells by Kupffer cells activated a mild inflammatory response associated with lymphocyte infiltration [23].

### 3.2. Toxicokinetics

Metabolically active tissues dependent on high rates of protein synthesis, such as cells of the gastrointestinal tract, hepatocytes, and proximal convoluted tubules of the kidney, are disproportionately affected by *A. phalloides* poisoning [26,27]. After ingestion, amanitins are absorbed rapidly through the intestinal epithelium and bind weakly to serum proteins. This enables rapid clearance from plasma into liver and kidney tissue. The liver is the first organ encountered after absorption and is the principal organ affected [1]. In the liver, amanitins are transported by Organic Anion Transporting Polypeptides (OATP) into hepatocytes, causing extensive centrolobular necrosis. In the kidneys, after glomerular filtration, amanitins are reabsorbed in the renal tubules, resulting in acute tubular necrosis [11,26].

Approximately 60% of absorbed α-amanitin is excreted into bile and then returned to the liver via enterohepatic circulation. Renal clearance is the preferred elimination route for amanitins, with reported concentrations of α-amanitin 6 to 90 times greater in the kidney than in the liver [3,26,28]. As they do not undergo metabolism, large amounts of amanitins are found in the urine. Due to rapid absorption, the toxins can be detected in urine within 90–120 min of mushroom consumption, with maximal excretion occurring in the first 72 h. Intestinal elimination has also been noted with case reports of human intoxication with *A. phalloides*, finding that a lethal amount (6.3 mg) of α-amanitin was eliminated via feces over 24 h [1,3,11].

### 3.3. Mechanisms of Toxicity

Amanitins directly interact with RNA polymerase II (RNAP II) in eukaryotic cells. Studies have demonstrated non-covalent nuclear inhibition of RNAP II when bound to amanitin [22,29]. This inhibits transcription, causing a progressive decrease in messenger ribonucleic acid (mRNA) levels, leading to deficient protein synthesis and, eventually, cell apoptosis and necrosis [4,11]. Experimental studies have identified RNAP II residues that interact with α-amanitin, which are located entirely in the bridge helix of the molecule and attach through a hydrogen bond to α-amanitin [30,31,32]. However, other studies have suggested that α-amanitin inhibits RNAP II by direct interference with the trigger loop, where direct substrate contact and nucleotide addition occur, therefore preventing the conformational change of RNAP II and inhibiting ribonucleic acid elongation [32,33].

In vitro studies have shown that liver injury in mammals by α-amanitin is driven by p53- and caspase-3-dependent apoptosis in hepatocytes [31,34]. The concentration required for p53 induction correlated with the concentration required to inhibit mRNA synthesis, suggesting a link between these two effects [3]. To further evaluate the role of p53 in transcription inhibition-mediated apoptosis, p53 knock-out HTC116 cells and wild-type cells were treated with α-amanitin for 24 h. The results showed that knock-out p53 cells were less sensitive to apoptosis. Knock-out p53/BAK mice also showed significant resistance to hepatotoxicity from α-amanitin exposure, and wild-type mice under the same conditions exhibited significant liver cell death [3,35,36].

Other proposed mechanisms of toxicity include α-amanitin acting in synergy with endogenous cytokines like tumor necrosis factor (TNF), which might cause cell damage by inducing apoptosis [1]. In vivo studies with mice have shown that, after administration of α-amanitin, concentrations of hepatic TNF-mRNA increased, and there was significant apoptosis of hepatocytes. However, in mice treated with anti-TNF antibodies, liver injury caused by α-amanitin was prevented [3,36,37]. TNF-α co-treatment has been shown to significantly increase lipid peroxidation caused by α-amanitin, which was prevented by silybin, suggesting that TNF-related toxicity may be related to the development of reactive oxygen species (ROS) [3,37]. Although some in vivo and in vitro studies have found some association between α-amanitin and the development of ROS, further investigation is needed to completely establish the pathophysiology of ROS in α-amanitin-associated toxicity [36,37].

## 4. Management of *Amanita phalloides* Poisoning

### 4.1. Diagnosis

Careful history and assessment of clinical presentation are key in the diagnosis of *A. phalloides* poisoning [38,39,40]. The association between mushroom ingestion and presentation is likely to be obscured by the delay between ingestion and symptom onset, as well as by the patient not associating significance to the consumption of mushrooms. Furthermore, due to the rarity of mushroom poisoning in clinical practice, clinicians will often have a low index of suspicion, which can further delay identification and treatment [2,41,42]. Detailed history includes establishing a description of the mushrooms, location of harvest, conditions of storage, preparation, and consumption, as well as the onset of symptoms of others who consumed the same mushrooms [11,12]. Determining the latency period of symptoms is important to address late toxicities such as liver and renal failure in a timely manner. Amatoxin ingestion should be suspected in patients reporting consumption of gilled mushrooms with a white or green cap or developing jaundice after an episode of presumed acute gastroenteritis [11,22,41]. The most reliable method of diagnosis remains the actual visualization of leftover mushrooms or microscopic identification or spores in cooked mushroom remains, vomit, urine, or feces by qualified mycologists who may be available through local poison centers [22,41].

Patients routinely undergo blood tests on presentation to the emergency department. This involves the assessment of electrolytes, renal function, liver chemistry, coagulation profile, and inflammatory markers, as well as early markers of severity such as lactate and serum LDH [22,43]. Routine stool and urine tests may be performed to rule out infection. Radiological assessment may include computed tomography of the abdomen in the context of abdominal pain and diarrhea or abdominal ultrasound to assess liver damage due to deranged liver chemistry [21,42,44].

Serum amatoxin levels are not routinely used in initial diagnosis but may be used as retrospective confirmation for *A. phalloides* poisoning [11]. The only specific laboratory test available is the detection of amatoxins in urine [19,45]. Qualitative detection of amatoxins in urine can be valuable since a confirmed diagnosis allows for earlier aggressive treatment, which can be key to reducing morbidity and mortality. Different methods of analysis are utilized, including high-performance liquid chromatography, Enzyme-Linked Immunosorbent Assay, and Radioimmunoassay, which are highly sensitive, without false negatives if performed in the first 48 h after ingestion [18,46,47]. After 36 h from the time of ingestion, the accuracy of the analysis is unreliable, and a negative result does not rule out amatoxin toxicity [28,46].

Yang et al. (2024) [47] recently developed a simple and rapid ultra-high-performance liquid chromatography (UPLC-MS-MS) method to identify amatoxins and phallotoxins in blood and urine samples. This was tested on real cases of *A. phalloides* poisoning with positive results, and blood and urine samples were obtained within 24 h of ingestion. This validated method did not need internal standards and large quantities of reagents while being more convenient and cost-effective than previous methods, enabling potential use in real-world clinical settings [47].

The Meixner test is used if a specimen of the ingested mushroom is available [48]. The test involves a catalyzed reaction using concentrated hydrochloric acid with the complex biopolymer lignin to form a blue product [3,48]. The mushroom juice is squeezed onto lignin-containing newsprint and dried. A drop of concentrated hydrochloric acid is then added. Development of a blue color reaction indicates positivity for amatoxins [3]. There is a high rate of false positives with the Meixner test, as mushrooms not containing Amatoxins may have a positive reaction. Hence, further testing is still required to confirm mushroom identity. As there are limited analytic options, suspected diagnosis needs to be made primarily on a clinical basis to enable early intensive treatment [3,48].

### 4.2. Strategies to Reduce Toxin Absorption

Gastric aspiration and lavage are usually performed with the administration of activated charcoal via a nasogastric tube [43]. Lavage within one hour of *A. phalloides* ingestion is most effective [5,49,50]. However, amatoxins are present in gastroduodenal fluids at least 60 h after toxin ingestion. Even if the toxin is unknown, activated charcoal is recommended if severe poisoning cannot be ruled out and there is no increased risk of aspiration [51]. Given the enterohepatic circulation of amatoxins, activated charcoal should bind toxins excreted via bile into the small intestine, and multiple doses of charcoal should reduce toxin absorption from the gastrointestinal tract [27,51]. Serial charcoal dosing as a continuous nasogastric drip or pulse dosing with 20–40 g every 3–4 h (for 24 h or more) has been most frequently quoted in the literature, and most local poison centers have their own guidelines [5,49]. Charcoal dosing is contraindicated in patients with an altered mental state, recent surgery, loss of airway protective reflexes, or severe gastrointestinal hemorrhage [49]. The use of activated charcoal has been associated with reduced peak values of bilirubin and INR with no significant reduction in peak AST or ALT levels, suggesting synthetic function recovery and preservation of liver function, likely reducing the risk of further end-organ damage [51,52].

### 4.3. Strategies to Increase Elimination of Absorbed Amatoxins

Amatoxins are detected in urine up to 4 days after ingestion. Strategies for enhancing renal clearance include intravenous (IV) fluid restoration and diuresis. Aggressive IV rehydration maintains renal function, corrects metabolic acidosis and electrolyte abnormalities, and is beneficial in mitigating irreversible hepatotoxicity [5,41,53]. Urinary output of 100–200 mL/h for 4–5 days is considered sufficient for improving renal elimination of amatoxins. Diuresis is recommended to meet this threshold, especially in the first 48 h after ingestion [5,40].

Biliary drainage has been trialed in this setting, but there is limited data on efficacy. Biliary drainage has been shown to reduce intestinal amatoxin absorption by more than 70% in dogs given lethal doses of amatoxins, improving survival [5,27,52]. Case reports in humans have shown that nasobiliary drainage or ultrasound-guided drainage and endoscopic biliary diversion have beneficial therapeutic effects in patients with amatoxin poisoning [5,52,54]. Considering the limited data on the efficacy of biliary drainage, further evaluation needs to be performed to assess its utility and role in amatoxin poisoning.

### 4.4. Extracorporeal Purification Procedures

Extracorporeal purification techniques, including hemodialysis, hemoperfusion, and plasmapheresis, have been used in the management of amatoxin toxicity. Although overall efficacy is limited, as amanitins are rapidly cleared from the blood [26], the use of extracorporeal purification, particularly plasma exchange, has improved transplant-free survival since their employment in the treatment of *A. phalloides* poisoning was introduced [26,55].

Plasmapheresis is an effective treatment for *A. phalloides* poisoning [5,55]. Plasmapheresis can remove amatoxins and metabolic wastes from the blood, as well as supply albumin, immunoglobulins, coagulation factors, and proteins to maintain an environment conducive to hepatocyte regeneration. The process involves the exchange of 1–3 L of patient plasma with a protein solution. The number of exchanges performed is determined by the changes in liver chemistry [55]. When plasmapheresis has been used in combination with other supportive therapies, the best results were obtained within the first 36–48 h after ingestion [5,55]. However, plasmapheresis is also effective when performed at later stages, particularly in situations of fulminant liver failure [26,55].

Hemodialysis has been used in amatoxin toxicity as a means of treating acute kidney injury, often leading to full recovery of renal function [5,56]. There have also been published reports of patients with confirmed *A. phalloides* ingestion undergoing hemodialysis and hemoperfusion in the absence of detected toxin [5,40]. Nonetheless, given the short half-life of toxin in plasma, the therapeutic effects of hemodialysis and hemoperfusion are suspected to be negligible in *A. phalloides* poisoning [5].

### 4.5. Artificial Liver Support Systems

There are two artificial liver support systems used to manage *A. phalloides* poisoning—the molecular adsorbent recirculating system (MARS) and Fractionated Plasma Separation and Absorption (FPSA). Both systems are albumin dialysis methods that selectively remove albumin-bound toxins and hydrophilic small molecules via a conventional dialysis loop most used in intensive care settings [57]. The removed toxins are suspected to be responsible for the progression of acute liver failure from hepatic encephalopathy, hepatorenal syndrome, cardiovascular failure, and/or the presence of an immuno-depressed state [3]. MARS uses an albumin-impermeable membrane against a continuously circulating loop with 20% human serum albumin passing through columns and a low-flux dialyzer connected to a secondary circuit, whereas FPSA allows a patient’s own albumin into contact with filter columns. Water-soluble substances are removed using a separate high-flux dialysis loop. MARS and FPSA remove protein-bound and water-soluble toxins and substances from the blood, thereby performing detoxifying functions of the liver [5,57,58].

MARS has also demonstrated benefits in addition to standard supportive therapy [59]. Although MARS removes toxins well, it provides smaller improvements to transaminase and bilirubin levels compared with plasmapheresis [5,60]. The use of MARS in conjunction with plasmapheresis showed improved efficacy in a small group of patients with *A. phalloides* toxicity and may be a more efficient option [5,19]. MARS has also been considered a suitable option as a bridge to liver transplantation in *A. phalloides* intoxication [5].

The use of FPSA has demonstrated favorable outcomes in patients with *A. phalloides* intoxication when used in addition to other supportive measures [61]. FPSA was found to efficiently clear amanitin, resulting in reduced hospital stays and less severe renal impairment [61]. In those needing liver transplants due to *A. phalloides* poisoning, FPSA was found to support a prolonged pre-surgical wait time for liver graft [62]. FPSA achieved significantly higher clearance of bilirubin, ammonia, and urea compared with MARS [5].

However, all studies evaluating MARS and FPSA to date have been retrospective studies employing small patient cohorts, with most findings not reaching statistical significance. Furthermore, these systems require highly subspecialized intensive care settings that are only available in very select centers with significant associated costs. In 2006, the cost of MARS alone in acute or chronic liver failure was EUR 14631, which would be similar to acute liver failure from *A. phalloides* poisoning but likely higher in today’s setting due to inflation [63]. Hence, more robust prospective data is required to fully assess their efficacy in *A. phalloides* poisoning with further cost-benefit analyses. It is also unlikely that artificial liver support systems will be more readily used due to limitations in access, clinician experience, and availability.

### 4.6. Drug Therapies

#### 4.6.1. Antibiotics

Benzylpenicillin is the most widely used agent against *A. phalloides* poisoning [3,5,34]. In vitro studies with human hepatocytes demonstrated that benzylpenicillin limited the cytotoxicity of amatoxins through potent inhibition of the OATP1B3 transporter [3,34]. In addition to other supportive measures, benzylpenicillin has shown increased efficacy, resulting in patient recovery in small cohort studies [5,39]. High doses are often used (40,000,000 units/day) in adults [49]. Unfortunately, benzylpenicillin commonly elicits allergic drug reactions with an incidence of 1–10%. Furthermore, there is a large sodium load with the administration of the antibiotic, which, in *A phalloides* poisoning, can lead to further electrolyte imbalances [34,49].

Ceftazidime has also been used in *A. phalloides* intoxication. The utility of ceftazidime is confounding in the literature, as it has always been administered concomitantly with silibinin [49,64], and further investigation is required to clarify its efficacy [3].

#### 4.6.2. Silibinin

Silibinin is the major active constituent of silymarin, which is an extract of milk thistle [64]. Experimental results show that silibinin inhibits amatoxin uptake by hepatocytes by competitive inhibition of the OATP system, specifically OATP1B3, during primary and enterohepatic circulation of toxin [26,34,65]. Silibinin may also inhibit TNF release in the injured liver. By stimulating protein synthesis, silibinin can enhance the regenerative capabilities of the liver by stimulating rRNA synthesis and preventing the development of liver and kidney failure [11,65]. In vitro models indicate that silibinin improves viability in α-amanitin hepatocytes with no added benefit from the addition of penicillin [29]. In approximately 1300 cases, overall mortality among *A. phalloides*-intoxicated patients treated with intravenous silibinin alone was better than those treated with penicillin [5,29,65].

Administration of silibinin within 48 h after *A. phalloides* ingestion was effective in preventing severe liver damage [49]. There is limited data on adequate dosing regimens, but some reports recommend loading at 5 mg/kg over an hour, then a continuous dose of 20 mg/kg daily until liver function and INR normalize [5,10,49]. Legalon^®^ SIL is a pharmaceutical formulation of silibinin with the active ingredient being silibinin-C-2′,3-dihydrogen succinate [65]. When parenteral formulations have not been available, oral Legalon has been utilized, although the efficacy of oral and parenteral formulations has not been compared in this setting [10,65]. Silibinin is the US Food and Drug Administration listed for use in *A. phalloides* poisoning through an emergency approval process [42].

#### 4.6.3. N-Acetylcysteine

N-acetylcysteine (NAC) has been used in *A. phalloides* poisoning for decades [5]. The role of NAC in this setting is not only as a “scavenger” of free radicals but also as a glutathione precursor as endogenous stores are depleted [5]. In experimental studies, NAC has demonstrated protective effects in human hepatocytes from α-amanitin-induced apoptosis [5,34]. Retrospective multidimensional multivariate statistical analysis of 2110 clinical cases of amatoxin poisoning showed that NAC was associated with higher survival in patients with amatoxin poisoning [63,66]. Other retrospective studies have also demonstrated the benefits of adding NAC as part of supportive therapy in *A. phalloides* poisoning [5,27,66]. In one analysis, the mortality rate was lower in patients receiving NAC with benzylpenicillin (4.4% with NAC vs. 18.7% without NAC) [38,66]. Dosing regimens are not standardized, but many studies followed paracetamol poisoning dosing guidelines [10].

#### 4.6.4. Indocyanine Green

Clustered, regularly interspaced short palindromic repeats (CRISPR) screens are widely used to identify genes or pathways involved in drug resistance or toxin mechanisms, which might lead to finding potential antidotes [34]. In one such study, CRISPR was utilized to identify potential US Food and Drug Administration-approved drug targets in α-amanitin toxicity. The N-glycan biosynthesis pathway and its main component, STT3B, were found to play a crucial role in α-amanitin toxicity. Depletion of STT3B significantly decreased the entrance of α-amanitin in human cells [34]. Indocyanine Green, a diagnostic reagent used in ocular and cerebral angiography, as well as hepatic function assessment, was found to be a potent STT3B inhibitor [34]. This study demonstrated Indocyanine Green’s efficacy in blocking toxic effects of α-amanitin in cells, liver organoids, and male mice and showed an overall increase in animal survival [34]. Although promising, further trials are required to determine if Indocyanine Green can be used as a potential antidote to α-amanitin.

#### 4.6.5. Glossy Ganoderma Decoction

Glossy Ganoderma Decoction (GGD) is a traditional Chinese medicine that contains polysaccharides, amino acids, terpenes, and steroids [67]. Twelve patients with acute *A. phalloides* poisoning were treated daily with GGD with supportive therapy. Mortality was greatly reduced compared with controls (supportive therapy alone) [67]. The number of patients enrolled in this study was too small to achieve significance; nonetheless, GGD may have potential use pending further investigations [5].

#### 4.6.6. Polymyxin B

Polymyxin B can potentially bind to RNAP II in the same binding site as α-amanitin, possibly preventing α-amanitin from binding RNAP II [68]. In an in vivo study, polymyxin B improved α-amanitin-induced liver and kidney injury, and administration significantly increased survival in α-amanitin-treated animals [68]. Further studies are needed to determine whether polymyxin B can be a potential pharmacological agent for treating *A. phalloides* poisoning [5,68].

## 5. Liver Transplantation

Liver transplantation is the only method of management with recognized survival benefits in patients with *A. phalloides* poisoning and poor prognosis [5]. However, the indication for transplant in this context has a degree of uncertainty [69]. The criteria used in making the decision to proceed to liver transplantation are summarized in Table 1. The King’s College criteria (Table 1) for non-paracetamol-induced liver failure have been most widely used in *A. phalloides* poisoning [3,5,53]. However, these criteria have limited applicability in the setting of *A. phalloides* poisoning [69]. Both King’s College criteria and Crichy’s criteria include hepatic encephalopathy as an important measure (Table 1). Small studies in *A. phalloides*-intoxicated patients have shown that hepatic encephalopathy was not present in all patients with fatal outcomes, and despite the absence of hepatic encephalopathy, they rapidly developed multi-organ failure [53]. Those who developed encephalopathy died soon after onset [53,69]. Ganzert and Escudié criteria do not include hepatic encephalopathy as a parameter [53,69].

The prognostic value of the different decision criteria pertaining to *A. phalloides* or mushroom-induced liver failure has been evaluated and compared [24,53]. The King’s College Criteria were most efficacious compared with Ganzert [69] and Clichy’s criteria for predicting fatal outcomes, defined as the need for transplant or death [53]. In this study, it was found that an interval of less than 8 h between toxin ingestion and the onset of diarrhea was significantly associated with the risk of fatal outcomes. Furthermore, all patients who developed diarrhea more than 8 h after toxin ingestion had non-fatal outcomes [53]. These findings led to the development of the Escudié criteria, which have shown superior sensitivity, specificity, and positive and negative predictive value in predicting fatal outcomes compared with the other criteria (Table 1) [70].

However, Ferreira et al. found that almost a third of cases in their cohort with fatal outcomes from *A. phalloides* poisoning did not have a prothrombin index of less than 10% of normal at day 3 after ingestion [71]. There have also been reports of rapid prothrombin time deterioration within a few hours, not days, in patients who developed acute liver failure from *A. phalloides* poisoning [72]. A more recent retrospective study in Korea [24] demonstrated that King’s College and Escudié’s criteria had 100% accuracy in predicting 28-day mortality. Furthermore, Escudié’s criteria were able to identify fatal cases earlier.

Although decision criteria (Table 1) provide a framework for the consideration of transplantation in *A. phalloides* poisoning, prognostic accuracy within a safe time interval for transplant is not consistently addressed by these criteria, as demonstrated by the differing findings of retrospective studies. Therefore, further prospective and randomized trials are needed to delineate the best and earliest prognostic markers in ALF induced by *A. phalloides*. The accuracy in predicting outcomes needs to also be widely reproducible. However, noting the rarity of *A. phalloides*-associated hepatotoxicity and barriers to early diagnosis, producing a large volume of prospective data in this area is challenging.

## 6. Summary

*A. phalloides* is identified as one of the most toxic mushrooms and has been associated with the most fatal cases of mushroom poisoning for centuries. A small number of cases of *A. phalloides* toxicity occur annually, and clinical recognition is often delayed due to lag between ingestion and symptoms, as well as presentation being very typical for acute gastroenteritis. The pathophysiology and mechanisms of toxicity, as currently understood, are intricate. RNAP II inhibition by α-amanitin has been proposed as the predominant mechanism of toxicity. Some therapeutic agents used in treating *A. phalloides* poisoning target this pathway, but there is little direct evidence regarding the mechanism and efficacy.

As it is an uncommon occurrence, there is very limited retrospective data on treatments and outcomes. This review found no prospective data on management strategies. Hence, the approach to treatment is varied as the evidence for different treatment modalities is based on small retrospective cohorts. Further clinical studies into the benefits of Indocyanine Green in α-amanitin toxicity would provide valuable information into the treatment of amanitin toxicity. Given the small cohorts of reported studies, the true efficacies of the different therapies have not been properly determined. Liver transplantation remains a mainstay of management with recognized survival benefits in acute liver failure patients with poor prognosis. It is evident that further large prospective studies are required to determine the optimal management of *A. Phalloides.* However, in the context of scarcity of occurrence and delays in recognition, obtaining such evidence remains very challenging.

## 7. Materials and Methods

Databases, including Medline, PubMed, Embase, Ovid, Cochrane Database, Google, and Google Scholar, were searched using keywords and MeSH terms to identify relevant peer-reviewed articles on *Amanita phalloides* from January 2000 to April 2024. Keywords included *Amanita phalloides*, amanitins, amatoxins, alpha amatin, hepatotoxicity, liver failure, liver transplantation, mushroom poisoning, and treatment. Articles were limited to the English language, and those suitable to answer the objectives of this review were included.

## Figures and Tables

**Table 1:** Decision criteria for emergency liver transplantation in patients with *Amanita phalloides* toxicity.

Clichy’s Criteria	King’s College Criteria for Non-Paracetamol Related ALF	Ganzert’s Criteria for Amatoxin Induced ALF	Escudié Criteria for Amatoxin Induced ALF
Decrease in Factor V < 30% of normal in patients >30 years of age. OR Decrease in Factor V < 20% of normal in patients <30 years of age. AND Grade 3–4 Encephalopathy	Coagulopathy: PT > 100 s (INR > 6.5) OR Any three of: Age: <10 years or >40 years; Duration from jaundice to onset of encephalopathy >7 days; PT > 50 s (INR > 3.5); Serum bilirubin > 300 μmol/L (17.5 mg/dL); Aetiology: non-A, non-B hepatitis, drug-induced or indeterminate cause of ALF.	Decrease in Prothrombin Index < 25% of normal at 3 to 10 days after *A. phalloides* ingestion. AND Serum Creatinine ≥106 µmol/L (1.2 mg/dL) within time period of PT rise	After toxin ingestion, onset of diarrhea <8 h OR Decrease in prothrombin index <10% of normal (approximately an INR > 6) ≥4 days after ingestion

ALF = acute liver failure, PT = prothrombin time, INR = International Normalised Ratio.

## Data Availability

No new data were created or analyzed in this study. Data sharing is not applicable to this article.
